# A 4-km long-term (1952–2025) daily hydrometeorological dataset for the western United States

**DOI:** 10.1038/s41597-026-07642-0

**Published:** 2026-06-25

**Authors:** Gavin D. Madakumbura, Ronnie Abolafia-Rosenzweig, Cenlin He, Jacob Jones, Qian He, Menaka Revel, Bowen Wang, A. Park Williams

**Affiliations:** 1 Department of Geography, University of California — Los Angeles, Los Angeles, CA USA; 2 Earth System Science Interdisciplinary Center, University of Maryland, College Park, MD USA; 3 Hydrological Sciences Laboratory, NASA Goddard Space Flight Center, Greenbelt, MD USA; 4 NSF National Center for Atmospheric Research, Boulder, CO USA; 5 Department of Civil and Environmental Engineering, Faculty of Engineering, University of Waterloo, Waterloo, ON Canada; 6 Department of Civil and Environmental Engineering, Massachusetts Institute of Technology, Cambridge, MA USA; 7 Department of Atmospheric and Oceanic Sciences, University of California — Los Angeles, Los Angeles, CA USA

## Abstract

This study introduces the **W**estern U.S. **Hi**gh-resolution **L**ong-term **D**ataset for **H**ydro**M**eteorology (WHiLD-HM), a 4-km, 74-year spatiotemporally continuous daily dataset spanning 1952–2025 for the western United States (WUS). WHiLD-HM is generated by driving the Noah-MP version 5.0 land surface model with observed precipitation, surface air temperature, downward shortwave radiation, and specific humidity, along with dynamically downscaled reanalysis-based surface air pressure, downward longwave radiation, and wind speed. The dataset encompasses key hydrometeorological variables, including surface energy and water fluxes. Cross-comparisons with observational datasets demonstrate the high temporal and spatial accuracy of WHiLD-HM. Case studies illustrate WHiLD-HM’s ability to capture long-term drying trends in WUS, as well as extreme drought and pluvial conditions. By extending coverage beyond the satellite era, WHiLD-HM enables high-resolution analyses of daily to multidecadal hydroclimatic variability and trends, as well as extremes, providing crucial insights for water resources and food security management and ecohydrological monitoring.

## Background & Summary

The western United States (WUS, the eleven westernmost states of the coterminous United States: Arizona, California, Colorado, Idaho, Montana, New Mexico, Nevada, Oregon, Utah, Washington, and Wyoming), home to 79 million people^1^ and a biodiversity hotspot^2^, consists of complex terrain and diverse climate zones^3^. WUS has been identified as the region with the most wildland conservation value^2,4^, and is the most water-stressed region in the coterminous United States^5,6^. Stakeholders from federal agencies to local communities in the WUS can gain substantial decision-support value from high-resolution, long-term datasets of surface water and energy fluxes that can aid quantification of hydroclimatic variability, improve basin-scale water budgets, and support water-resource and ecosystem planning across diverse landscapes^7,8^.

The temporal coverage of high-resolution water and energy flux and state data becomes an important factor when attempting to identify and understand longer shifts in climate beyond short-term variability^9^. The WUS hydroclimate is tightly coupled with natural climate variability at subseasonal to seasonal timescales such as the Madden-Julian Oscillation^10^, multiyear timescales such as the El Niño-Southern Oscillation^11^, and decadal timescales such as the Pacific Decadal Oscillation^12^. The region also has experienced longer-term hydroclimatic shifts due to greenhouse gas and aerosol emissions^13^. High-resolution water and energy flux data that also span multiple decades, beyond the satellite era (circa 1979), can provide crucial insights into hydroclimatic variability across temporal scales and inform local to regional adaptive management strategies for water resources planning in the face of ongoing environmental change.

This paper presents the University of California, Los Angeles (UCLA)-National Science Foundation National Center for Atmospheric Research (NSF NCAR) collaborative **W**estern U.S. **Hi**gh-resolution **L**ong-term **D**ataset for **H**ydro**M**eteorology (WHiLD-HM), a 4-km, spatiotemporally continuous 74-year (1952–2025) daily dataset for WUS, developed using a widely used, state-of-the-art, physically-based land surface model, Noah-MP v5.0^14^. A list of key variables represented in the dataset is available in Table 1. WHiLD-HM offers several advantages over existing and widely used hydrometeorological datasets such as Livneh^15^ (spatial resolution 6-km for 1915–2018) and North American Land Data Assimilation System project phase 2 (NLDAS-2)^16^ (spatial resolution 12-km for 1979–present). Specifically, WHiLD-HM provides a finer spatial resolution, uses higher-quality observational climate forcing data, and uses a more advanced, state-of-the-art land surface model than that used for NLDAS-2 (which generally relies on forcing from the North American Regional Reanalysis^17^ with land surface model physics that have not been updated recently^16^). WHiLD-HM also has a substantially longer temporal extent than NLDAS-2, extending through near-present. This enables more detailed and consistent analyses of hydroclimatic variability across the region. We first present a summary of the dataset and an application for understanding pre-satellite era hydrological extremes, followed by the methods used to create the dataset.

**Table 1:** Description of key variables included in the WHiLD-HM.

Variable	Category	Long name	Units
FSA	Radiation & energy balance	Total absorbed SW radiation	W m^−2^
FIRA	Radiation & energy balance	Total net LW radiation to atmosphere	W m^−2^
GRDFLX	Radiation & energy balance	Heat flux into the soil	W m^−2^
HFX	Radiation & energy balance	Total sensible heat to atmosphere	W m^−2^
LH	Radiation & energy balance	Total latent heat to atmosphere	W m^−2^
ECAN	Evapotranspiration	Canopy water evaporation rate	mm s^−1^
ETRAN	Evapotranspiration	Transpiration rate	mm s^−1^
EDIR	Evapotranspiration	Direct soil evaporation rate	mm s^−1^
ALBEDO	Surface properties	Surface albedo	—
UGDRNOFF	Runoff	Accumulated underground runoff	mm
SFCRNOFF	Runoff	Accumulated surface runoff	mm
T2MV	Near-surface temperature	2-m air temperature (vegetated)	K
T2MB	Near-surface temperature	2-m air temperature (bare ground)	K
SOIL_T	Soil state	Soil temperature	K
SOIL_M	Soil state	Volumetric soil moisture	m^3^ m^−3^
SOIL_W	Soil state	Liquid volumetric soil moisture	m^3^ m^−3^
SNOW_T	Snow state	Snow layer temperature	K
SNOWH	Snow state (surface)	Snow depth	m
SNEQV	Snow state (surface)	Snow water equivalent	mm
FSNO	Snow state (surface)	Snow cover fraction on the ground	—
QSNOW	Snow & precipitation fluxes	Snowfall rate to the ground	mm s^−1^
QRAIN	Snow & precipitation fluxes	Rainfall rate to the ground	mm s^−1^
ACSNOW	Snow & precipitation fluxes	Accumulated snowfall	mm
ACSNOM	Snow & precipitation fluxes	Accumulated snow melt water	mm
QSNBOT	Snow & precipitation fluxes	Water flux out of snow bottom	mm s^−1^
QMELT	Snow & precipitation fluxes	Snow melt due to phase change	mm s^−1^
FPICE	Snow & precipitation fluxes	Fraction of precipitation falling as snow	—

**Fig. 1:**
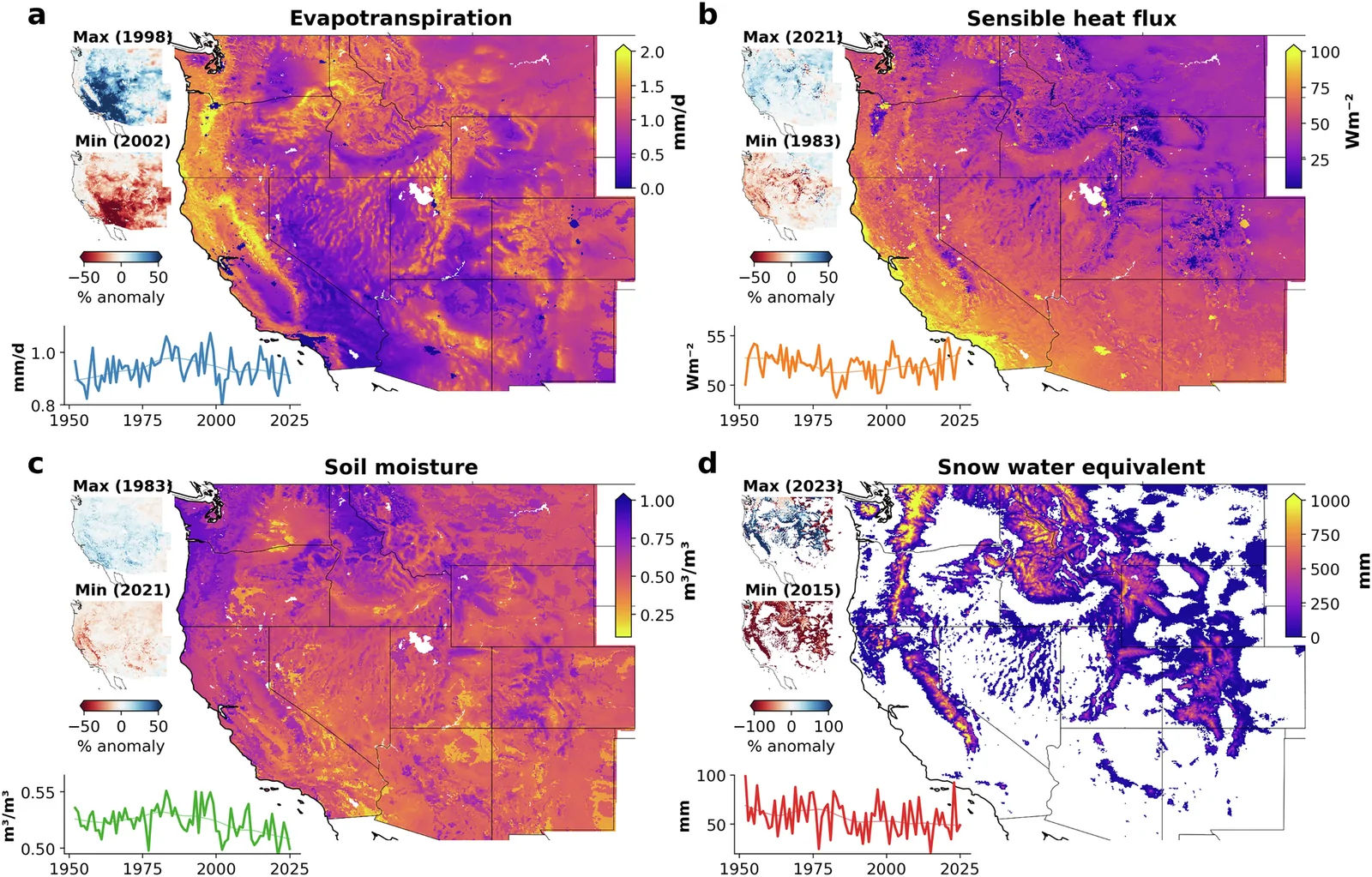
Climatology, long-term time series, and spatial anomalies of extremes during 1952–2025 for (**a**) evapotranspiration, (**b**) sensible heat flux, (**c**) 0–2 m depth integrated summer (June–August) soil moisture, and (**d**) April 1st snow water equivalent. Time series inset of each variable shows the WUS average values for each water year in (**a**) and (**b**), summer for (**c**), and April 1st for (**d**), along with the nonlinear locally weighted scatterplot smoothing trend. Spatial insets of each variable show the spatial maps of the percentage anomaly of the maximum and minimum events identified from the time series.

Figure 1 shows maps of long-term means of evapotranspiration (ET, Fig. 1a), sensible heat flux (SH, Fig. 1b), summer soil moisture (SM, Fig. 1c), and April 1st snow water equivalent (SWE, Fig. 1d), as well as time series for the WUS, and spatial maps of minimum and maximum for each variable. The dataset captures high-resolution spatial features across the domain. The long-term time series can represent previously documented long-term drying trends^18,19^ that would be difficult to identify in datasets with a shorter temporal coverage because low-frequency natural climate variability^20^ can confound this signal. A decrease in SM (Fig. 1c) represents previously documented long-term drying and the megadrought conditions in the WUS. In a dataset beginning in the late 1970s, the shift from the wet 1980s–90s to the 2000s megadrought may appear as a monotonic drying trend, whereas a longer-term WHiLD-HM record shows both periods to be partly transient rather than indicative of a persistent shift. The drying trend, particularly since the early 1980s, corresponds to a decrease in ET (Fig. 1a), reflecting moisture limitations. This, in turn, contributes to an increase in SH (Fig. 1b), as the energy that would otherwise be utilized by ET (or latent heat flux) is increasingly dissipated as SH^21^. April 1st SWE shows a slight decrease averaged across the WUS (Fig. 1d). Regarding extreme water years, the drought year 2021^22^ stands out as the SM minimum and SH maximum during the record. The years 1983 and 2023 correspond to the maximum values of SM and SWE, respectively, highlighting extreme pluvial conditions in the region^23^. The lowest SWE year is 2015, corresponding to the extreme 2012–2015 drought in California^24^. The year 1998 is the maximum ET year, possibly related to the 1997/98 extreme El Niño conditions^25^.

**Fig. 2:**
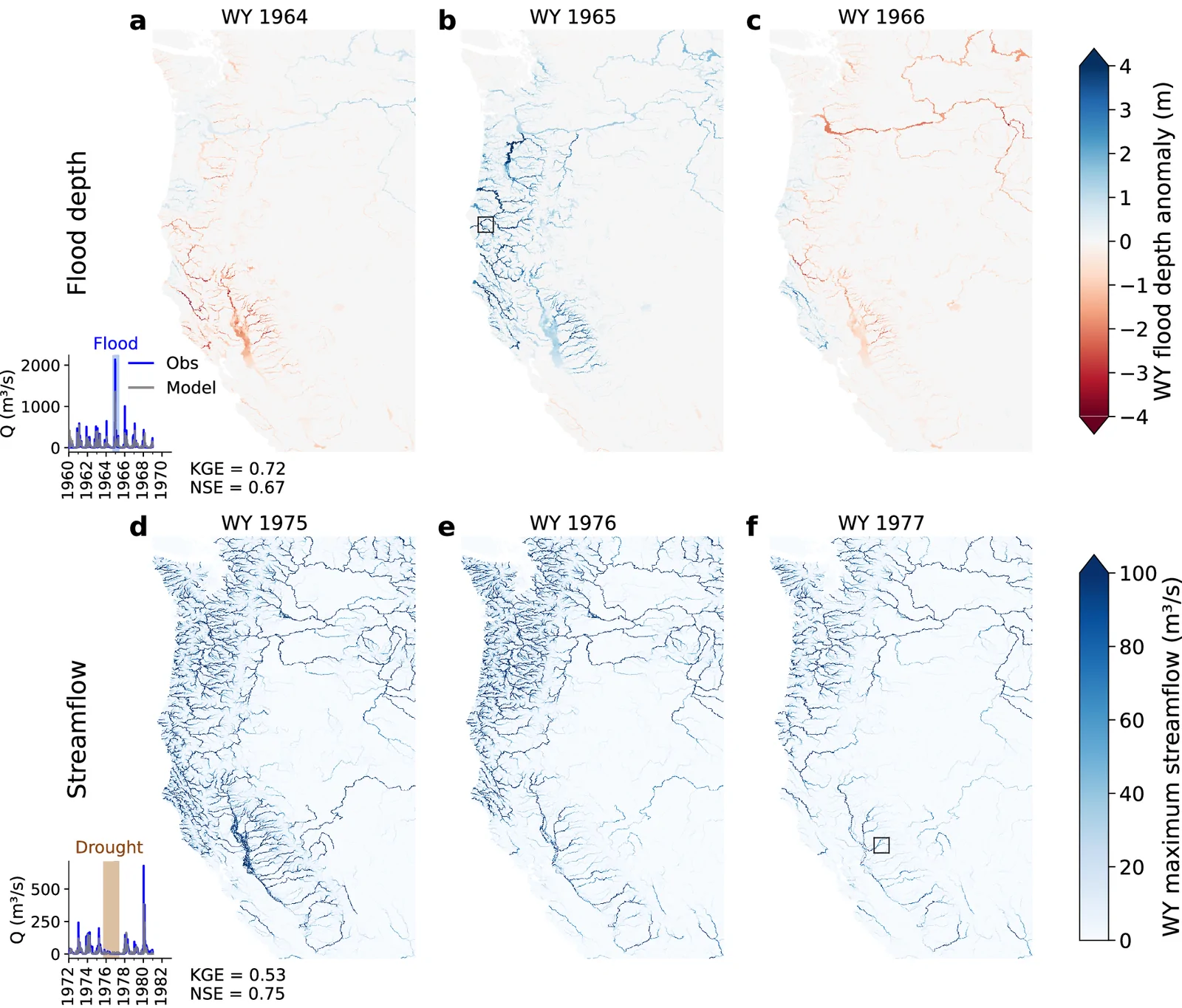
Application of WHiLD-HM to investigate extreme pluvial and drought events. (**a**–**c**) Anomaly of water year maximum floodplain depth during the 1964/65 extreme flood in northern California/Pacific Northwest. (**d**–**f**) Water year maximum streamflow during the 1976/77 extreme drought in California. The black boxes in panels (**b**) and (**f**) indicate example regions where the observed and modeled streamflow values shown in the insets are estimated.

Next, we present an application of the WHiLD-HM to understand hydrological extremes. We selected the December 1964 – January 1965 extreme floods in northern California and the Pacific Northwest^26^ as the pluvial extreme, and the 1976/77 extreme drought in California^27^ as the dry extreme. Both of these events occurred prior to the satellite era and lack spatially and temporally complete observations. We use the WHiLD-HM to force a high-resolution hydrodynamic floodplain model (see Methods for details) to investigate the hydrological conditions during these events. During the 1964/65 flood event, positive water year maximum flood depth anomalies can be seen across the region, with values upward of 4 m in Oregon and northern California (Fig. 2a–c). During the 1976/77 drought event, the water year streamflow values became extremely low (Fig. 2d, inset). Streams in California and the interior Pacific Northwest experienced extremely low flows during this event, and some even disappeared from the map at the considered spatial resolution (Fig. 2e,f). The interior Pacific Northwest experienced more severe drought conditions during the second year. During these flood and drought extreme events, regions we highlight as affected were documented in the past with regional accounting^27,28^. Using WHiLD-HM, we can estimate the spatial extent, intensity, and severity of hydrological extremes, demonstrating the uniqueness and usefulness of the dataset.

## Methods

### Land surface model

Land surface model simulations are generated across the WUS using the modernized Noah-MP version 5.0^14^ (https://github.com/NCAR/noahmp), a physically-based land surface model that simulates land surface processes through comprehensive representations of energy, water, and biochemical cycles^14,29^. Noah-MP is widely used in research and operational applications such as the National Water Model^30^, NASA Land Information System^31^, and NOAA Unified Forecast System^32^. The model computes sensible and latent heat fluxes by solving terrestrial energy budgets that incorporate radiative absorption and reflection, precipitation-advected heat transfer, and ground heat fluxes. Sensible heat includes fluxes from canopy, snowpack, and soil surfaces. Latent heat includes snowpack sublimation, canopy snow sublimation, bare ground evaporation, canopy water evaporation, and plant transpiration. For snow dynamics, Noah-MP has a multi-layer snowpack physics representation that dynamically tracks ice and liquid water content. Vertical distribution of soil moisture is simulated using one-dimensional Richards’ equation with multiple parameterization options for surface and subsurface runoff as well as groundwater processes. More information on Noah-MP can be found in ref. ^33^.

### Forcing datasets

Daily 4-km (1/24°) atmospheric forcing for 1951–2025^34^, developed in ref. ^35^, is used to force Noah-MP. This dataset incorporates daily maximum and minimum surface temperatures and precipitation totals from the 1/24° NOAA nClimGrid daily dataset^36,37^.

Daily vapor pressure (ea) is estimated by applying the Clausius–Clapeyron relation to 1/24° PRISM AN81d daily mean dew point for 1981–present^38,39^. Values are extended back to 1951 using the 9-km dynamically downscaled ERA5 dataset^40^ using the Weather Research & Forecasting Model (ERA5-WRF)^41,42^, which spans September 1950–March 2025. Daily mean specific humidity (q) and surface pressure (p) from the ERA5-WRF dataset are used to estimate vapor pressure as ea = pq/(0.622 + 0.378q), bilinearly interpolated to 1/24° resolution, and quantile-mapped so that daily (ea) distributions match PRISM during 1981–2020 for each grid cell and calendar month.

Wind velocity is based primarily on the 4-km WTK-LED dynamically downscaled ERA5 wind reanalysis for 2001–2020^43,44^, which has demonstrated skill across the WUS^45^. Daily means are calculated from hourly 10-m wind speeds, nearest-neighbor interpolated to the 1/24° grid, and converted to 2-m equivalent wind speeds by multiplying by 0.748, following FAO56 Penman–Monteith methodology^46^. The record is extended to 1951–March 2025 using ERA5-WRF daily mean 10-m wind, bilinearly interpolated to 1/24° and similarly height-adjusted, and extended through December 2025 using gridMET 2-m daily mean wind, which covers 1979–present^8,47^. ERA5-WRF and gridMET wind fields are quantile-mapped to WTK-LED over 2001–2020 for each grid cell and calendar month.

Downward shortwave radiation is taken primarily from the PRISM AN81d daily 1/24° observation-based solar radiation dataset for 1981–present. Solar radiation is extended back to 1951 using ERA5-WRF daily mean downward solar radiation by quantile-mapping to PRISM distributions during 1981–2020.

Downward longwave radiation is taken from ERA5-WRF as the primary source for 1951–March 2025, with fields bilinearly interpolated to 1/24° resolution. For April–December 2025, hourly NLDAS-2 forcing data at 1/8° resolution, available from 1979–present^16^, are aggregated to daily means, bilinearly interpolated to 1/24°, and quantile-mapped to match ERA5-WRF distributions during 1981–2020.

### Model spin-up and the simulation for 1952–2025

For model spin-up, we construct a pseudo-daily climate dataset covering 1940–1950 using monthly climatological data from our daily meteorological forcing dataset. We first extract daily anomalies for each variable by removing the monthly seasonal cycle from the forcing dataset for the year 1951, the first year of the forcing dataset. These daily anomalies are then repeatedly superimposed onto each year within the 1940–1950 period to generate the pseudo-daily climate dataset. Hourly forcing data are created from this pseudo-daily dataset using the same procedures applied to the actual forcing data. The model spin-up is conducted for 1940–1950 using the pseudo forcing data and for 1951 using actual observationally-based forcing data. This approach allows sufficient time for the model to initialize long-timescale processes (such as deep soil moisture and temperature) and establishes realistic initial conditions for the subsequent full simulation beginning in January 1952. WHiLD-HM is created from a simulation from October 1, 1951, to September 30, 2025 using the initialized conditions established through the spin-up process.

### Observational data used for comparison

We compare WHiLD-HM ET, SH, SM, SWE, and runoff against observational estimates. For ET, we compare simulated values with the daily gridded reconstructions from the 0.1° Global Land Evaporation Amsterdam Model (GLEAM) version 4.2a^48^ dataset covering 1980–2024 and the 0.25° FLUXCOM-X-BASE observationally-based daily ET^49^ dataset for 2001–2021. For SH, we use FLUXCOM observational reconstructions^50^ at an 8-day temporal resolution and 0.0833° spatial resolution for 2001–2015. We compare simulated surface SM anomalies with retrievals from the Soil Moisture Active Passive (SMAP) Enhanced L3 Radiometer Daily 9-km dataset^51^ for 2015–2025. SM evaluations are conducted in anomaly space, consistent with previous research^52^, acknowledging the limitation that simulated soil moisture from land surface models is generally best interpreted as a model-specific soil wetness index rather than a true representation of soil water volume^53^. We compare simulated SWE with the National Snow and Ice Data Center’s Snow Data Assimilation System (SNODAS) daily 1-km data^54^ for 2003–2024. For runoff, we compare WHiLD-HM with monthly mean streamflow observations from the Geospatial Attributes of Gages for Evaluating Streamflow II (GAGES-II) dataset^55^ for water years 1980–2020. Of these gauges, 434 are designated as reference basins, representing minimally disturbed catchments with limited human influence^55^. WHiLD-HM gridded runoff is spatially aggregated over each gauge’s upstream contributing area and resampled to monthly means for direct comparison with observed streamflow, which is converted to mm d^−1^ using basin area. The above observational datasets are selected to achieve a balance between spatiotemporal resolution and coverage. For grid-wise comparisons, following previous literature^56^, WHiLD-HM data are bilinearly resampled to match the coarse-resolution grids of GLEAM ET, FLUXCOM SH, and SMAP SM datasets. SNODAS SWE data are bilinearly interpolated to the WHiLD-HM grid.

### Hydrodynamic modeling for the case study and streamflow validation

To demonstrate the utility of WHiLD-HM, we simulate streamflow and floodplain conditions for the pre-satellite period (1952–1980) using the Catchment-based Macro-scale Floodplain global hydrodynamic model, version 4.2 (CaMa-Flood)^57^. River network and topography are obtained from MERIT DEM^58^ and MERIT Hydro^59^, respectively. CaMa-Flood is forced by WHiLD-HM surface and subsurface runoff and solves the local inertial approximation of the shallow water equation to model streamflow, river water levels, and floodplain inundation. CaMa-Flood simulations are conducted at 0.0167° spatial resolution. As a case study, we analyze conditions during the 1964/65 extreme flood in northern California and Pacific Northwest^26^, and the 1976/77 extreme drought in California^27^.

To validate the CaMa-Flood simulations, we use the U.S. Geological Survey National Water Information System gauge-based daily streamflow database^60^. Widely used evaluation metrics for hydrological applications, Kling-Gupta efficiency^61^ and Nash-Sutcliffe efficiency^62^, are calculated for the validation. For representative regions in the Pacific Northwest and California for the pluvial and drought events, respectively (Fig. 2b,f), we find high *r*-values (0.82 for Pacific Northwest, 0.92 for California) and above-average NSE (0.67, 0.75) and KGE values (0.72, 0.53).

## Data Records

WHiLD-HM is publicly available through an unrestricted data repository hosted by Harvard Dataverse (https://doi.org/10.7910/DVN/X7EYAN)^63^. The complete dataset comprises daily data for 1952–2025 and is stored in NetCDF format. Table 1 summarizes the key variables, while the full set of variables is included in the archived data files and provided as a separate variable list in the data archive. Daily WHiLD-HM data files are organized by water year in NetCDF files named WY_YYYY.nc. Each file contains daily data for the corresponding water year. Daily accumulated variables, such as surface runoff, subsurface runoff, snowfall, and snowmelt, are stored as daily totals. Other variables are daily average values by taking the mean of hourly data. Modeled daily soil moisture and soil temperature are provided for four soil layers (0–10 cm, 10–40 cm, 40–100 cm, and 100–200 cm).

In addition to the daily WHiLD-HM data files, the repository also provides the model configuration file (namelist.hrldas) and the static setup file (SETUP_WUS.nc) containing time-invariant land surface parameters and initial land surface conditions, which are summarized in Table 2.

**Table 2:** Initial land surface conditions.

Variable	Category	Long name	Units
CANWAT	Canopy water state	Canopy water	kg m^−2^
ZS	Soil vertical structure	Depths of centers of soil layers	m
DZS	Soil vertical structure	Thicknesses of soil layers	m
HGT	Topography	Terrain height	m
ISLTYP	Land surface classification	Dominant soil category	—
IVGTYP	Land surface classification	Dominant vegetation category	—
LAI	Vegetation properties	Leaf area index	m² m^−2^
XLAND	Land–water mask	Land mask (1 = land, 2 = water)	—
SNOW	Snow properties	Snow water equivalent	kg m^−2^
TMN	Temperature boundary	Soil temperature at lower boundary	K

The daily forcing dataset used to drive WHiLD-HM is archived separately at an unrestricted data repository hosted by Zenodo^34^ (https://doi.org/10.5281/zenodo.19636838).

## Technical Validation

**Fig. 3:**
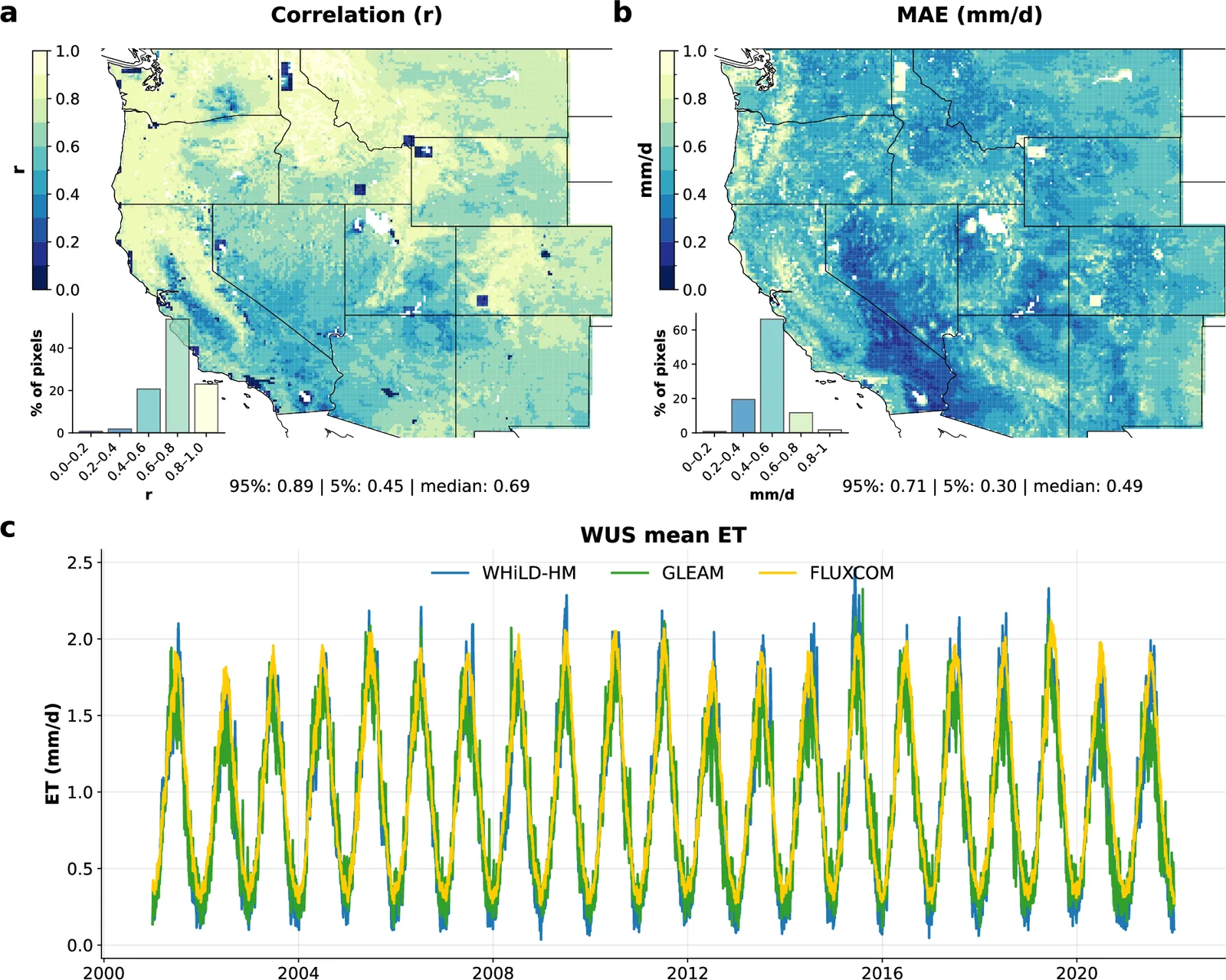
Evapotranspiration (ET, mm/d) comparison of WHiLD-HM with GLEAM and FLUXCOM-X-BASE. Grid-wise (**a**) Pearson correlation (*r*), (**b**) mean absolute error (MAE) between WHiLD-HM and GLEAM daily ET during 1980–2024 on the GLEAM grid (0.1° spatial resolution). Insets show the distribution of *r* and MAE in each panel. (**c**) Daily WUS average ET time series from WHiLD-HM, GLEAM, and FLUXCOM-X-BASE for the common period among datasets.

Figure 3 shows the comparison between WHiLD-HM and GLEAM ET for the period 1980–2024 at a daily timescale (Fig. 3a,b). Overall, the distribution of *r*-values indicates a high agreement between WHiLD-HM and GLEAM, where the median *r*-value is about 0.69 (*p* < 0.01). High *r*-values can be seen over the forests (California and Pacific Northwest), grasslands (Montana, Idaho, Wyoming, and Colorado), and croplands in Montana, Wyoming and Colorado. Irrigated croplands in California’s Central Valley and Washington are among the regions with the lowest *r*-values, likely attributable largely to the fact that Noah-MP simulations do not include irrigation. Arid regions such as deserts where the surface is barren or sparsely vegetated also show low correlations, potentially due to low temporal variability in ET in these regions. The distribution of mean absolute error (MAE) has a median value of 0.49 mm/d. The largest MAE values can be seen in the northern part of the Central Valley in California and parts of eastern Oregon and Washington. Figure 3c shows daily WUS average ET time series for WHiLD-HM, GLEAM, and FLUXCOM-X-BASE, for the period 2001-01-01 to 2021-12-31, the period when data are available for all three products. Broad agreement among the three datasets can be seen (WHiLD-HM–GLEAM: *r* = 0.96, p < 0.01; WHiLD-HM–FLUXCOM-X-BASE: *r* = 0.97, p < 0.01), especially in the seasonal cycle. Deseasonalized correlations between WHiLD-HM and GLEAM is 0.73 (p < 0.01), and WHiLD-HM and FLUXCOM-X-BASE is 0.68 (p < 0.01). WHiLD-HM has a larger temporal variability, namely larger daily anomalies, in periods with seasonal minimum and maximum ET.

Although GLEAM ET is largely informed by satellite observations, it is a model-reliant product based on climate, vegetation, and other variables, and FLUXCOM-X-BASE is a machine-learning-based gap-filled product, and thus discrepancies between WHiLD-HM and reference datasets are likely at least partly attributable to errors within each dataset.

**Fig. 4:**
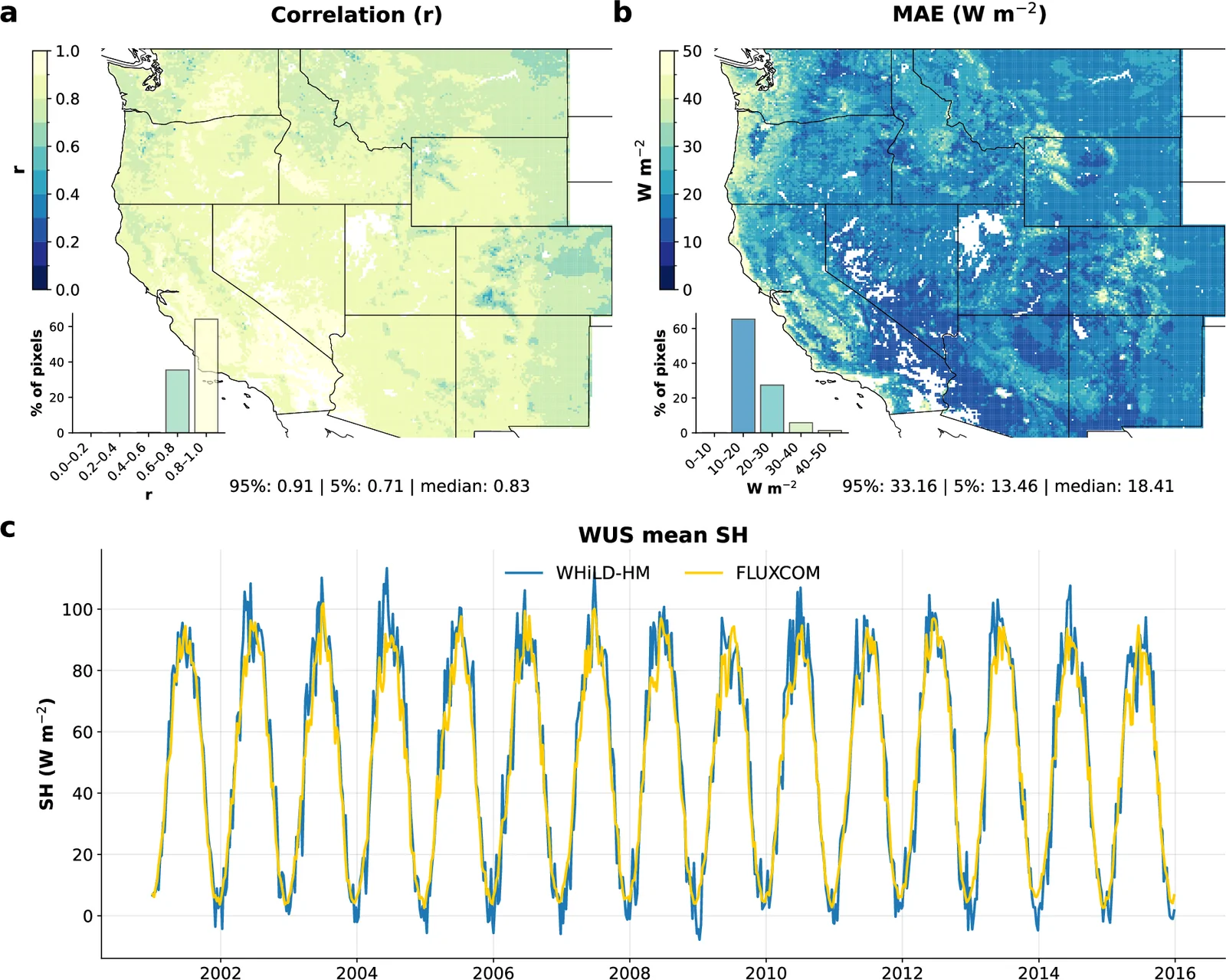
Sensible heat flux (SH, Wm^−2^) comparison between WHiLD-HM and FLUXCOM. Grid-wise (**a**) Pearson correlation (*r*), (**b**) mean absolute error (MAE) between WHiLD-HM and FLUXCOM 8-day SH during 2001–2015 on the FLUXCOM grid (0.0833° spatial resolution). Insets show the distribution of *r* and MAE in each panel. (**c**) 8-day WUS average SH time series from WHiLD-HM and FLUXCOM.

Figure 4 shows the comparison of 8-day SH between WHiLD-HM and FLUXCOM for the period 2001 to 2015. Very high *r*-values can be seen across the region, with values mainly ranging between 0.6–0.95, with the median value of 0.83. Forests in the Pacific Northwest, California’s Central Valley, and the Pacific coast stand out as regions with a large MAE, suggesting spatially heterogeneous uncertainty sources. WUS-averaged time series between the two datasets (Fig. 4c) show good agreement (*r* = 0.97, p < 0.01; deseasonalized *r* = 0.61, p < 0.01).

**Fig. 5:**
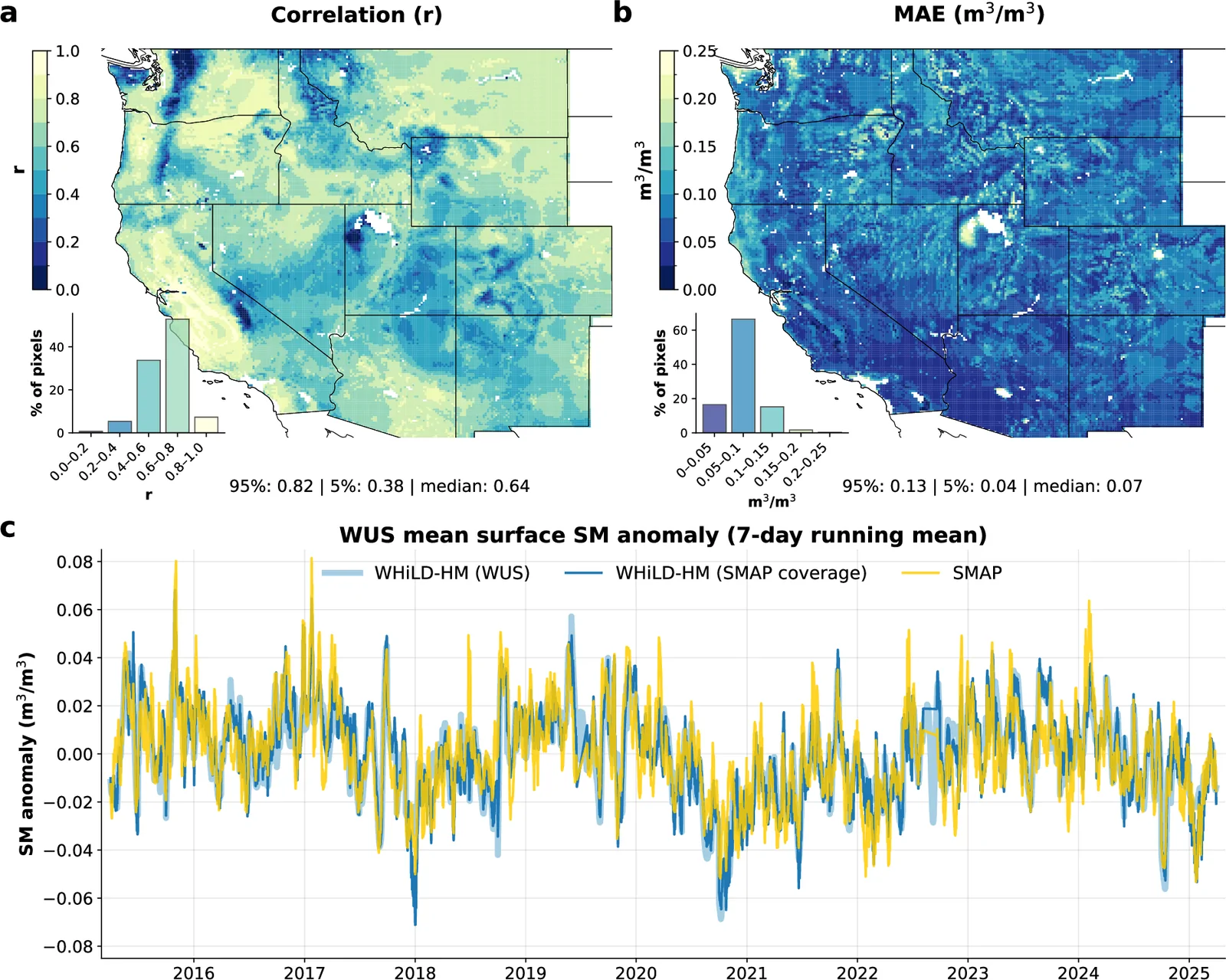
Surface soil moisture anomaly (SM, m^3^/m^3^) comparison between WHiLD-HM and SMAP. Grid-wise (**a**) Pearson correlation (*r*), (**b**) mean absolute error (MAE) between WHiLD-HM and SMAP daily SM during 2015-04-01 to 2025-03-31 on the SMAP grid (9-km spatial resolution). Insets show the distribution of *r* and MAE in each panel. (**c**) 7-day running mean of WUS average surface SM anomaly time series from WHiLD-HM and SMAP for the region covered by SMAP on each day [WHiLD-HM (SMAP coverage)] and for the whole WUS [WHiLD-HM (WUS)]. Anomalies for each time series are estimated by subtracting the monthly climatology from the corresponding daily values.

Figure 5 shows the comparison between daily surface soil moisture from WHiLD-HM and SMAP from 2015-04-01 to 2025-03-31. Correlations have a median of 0.64 across the domain. The lowest *r*-values, and therefore the largest disagreement between the two datasets, are observed over high elevation regions with snow, where SMAP retrieval accuracy can be negatively impacted by snow and frozen soil^51^. Arid desert regions in California and Nevada, and the Four Corners region (Arizona, New Mexico, Utah, and Colorado), where the climatological surface SM is low (Fig. 1), also show lower *r*-values. WUS average daily surface SM anomalies between the two datasets show a strong agreement (*r* = 0.83, p < 0.01). Dry periods where the SM anomalies are negative, such as 2017–2018 and 2021, are well-captured by WHiLD-HM, supporting high utility for representing drought conditions. WHiLD-HM underestimates periods with largely positive SM anomalies. Note that due to satellite overpass timing, the SMAP L3 product is not available for the whole WUS to estimate WUS average soil moisture, a limitation of SMAP, while WHiLD-HM offers the advantages of full spatial coverage.

**Fig. 6:**
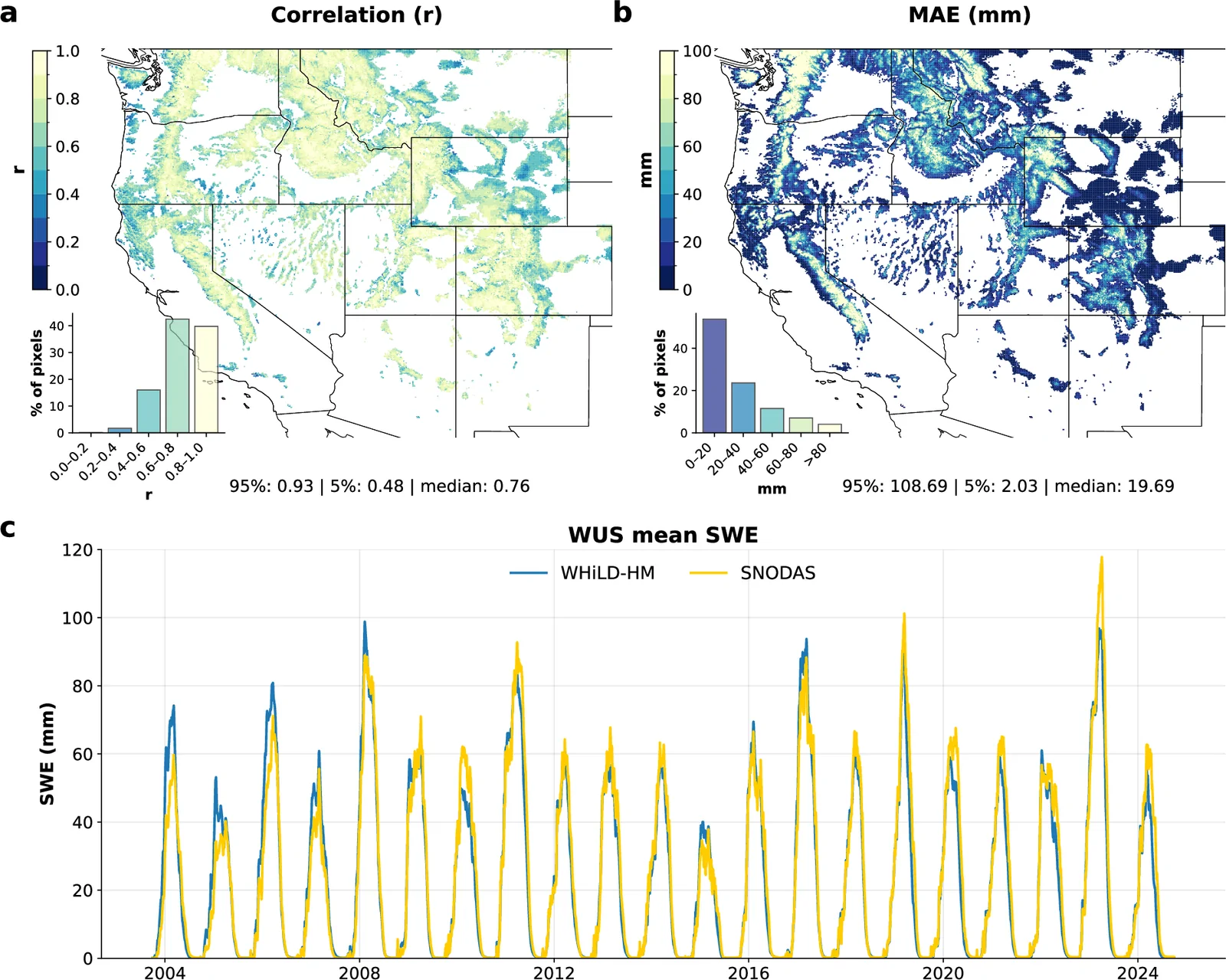
Snow water equivalent (SWE, mm) comparison between WHiLD-HM and SNODAS. Grid-wise (**a**) Pearson correlation (*r*), (**b**) mean absolute error (MAE) between WHiLD-HM and SNODAS daily SWE during 2003-09-30 to 2024-09-30 on the SNODAS grid (1-km spatial resolution). Insets show the distribution of *r* and MAE in each panel. (**c**) Daily WUS average SWE time series from WHiLD-HM and SNODAS.

Figure 6 shows the comparison between daily WHiLD-HM and SNODAS SWE during 2003-09-30 to 2024-09-30. The distribution of the *r*-values (Fig. 6a) shows high correspondence between the two datasets, with a median *r*-value of 0.76. Low *r*-values are mainly present in the lower elevations, where SWE is smaller and often ephemeral (Fig. 1d). MAE values are largest where the climatological SWE is highest (Fig. 1d). Figure 6c shows the daily WUS average SWE from the two datasets, highlighting strong agreement (*r* = 0.97, p < 0.01; deseasonalized *r* = 0.91, p < 0.01).

**Fig. 7:**
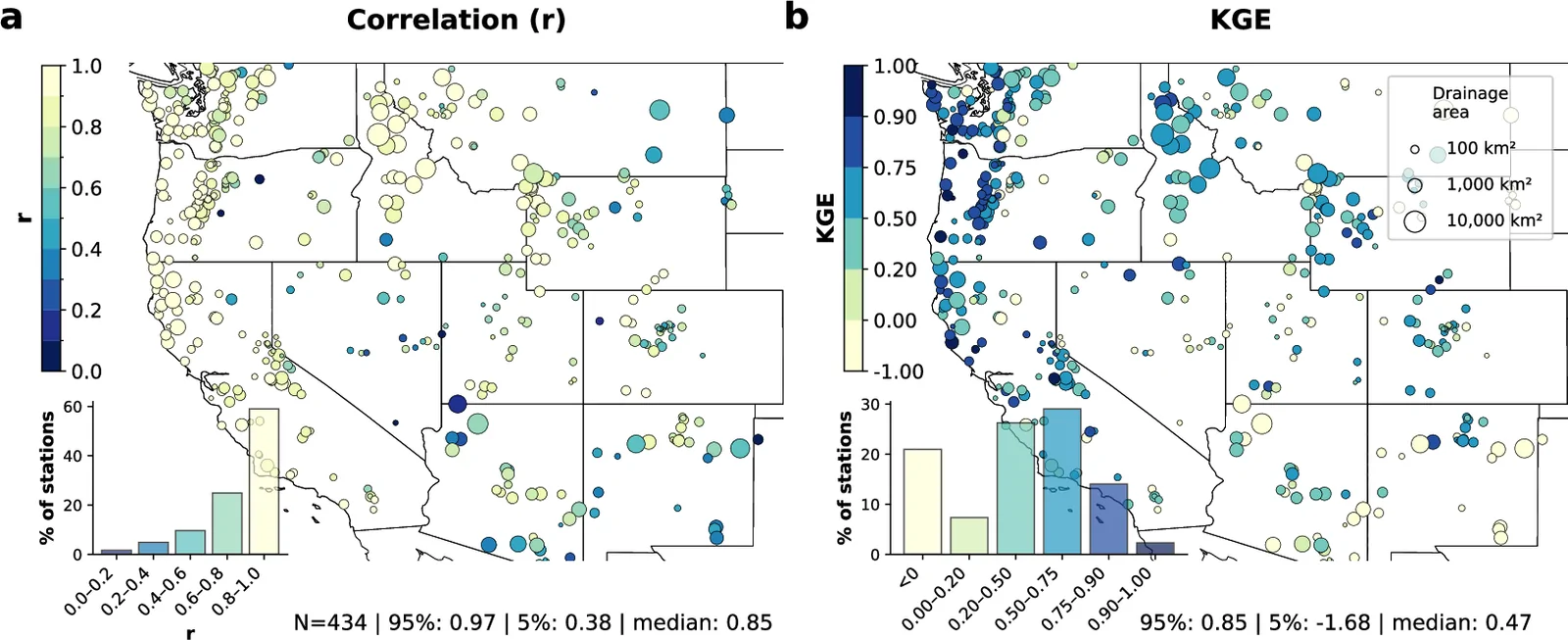
Monthly streamflow comparison between WHiLD-HM and GAGES-II reference basins (N = 434) across the WUS during water years 1980–2020. Station-wise (**a**) Pearson correlation (*r*) and (**b**) Kling-Gupta Efficiency (KGE) between WHiLD-HM and GAGES-II monthly streamflow. Insets show the distribution of *r* and KGE in each panel. Marker size encodes drainage area (legend in panel b). WHiLD-HM gridded runoff is aggregated by spatial summation over the upstream contributing area of each GAGES-II gauge to produce a basin-mean runoff series, then resampled to monthly mean for the comparison.

Figure 7 shows the comparison between monthly basin-mean runoff from WHiLD-HM and observations at 434 GAGES-II^55^ reference basins across the WUS from October 1979 to September 2020. The reference basin subset comprises catchments least disturbed by human activities, isolating the model’s intrinsic skill from anthropogenic regulation. Correlations have a median of 0.85 across the domain, and 74% of basins exceed *r* = 0.7. KGE has a median of 0.47, with 45% of basins reaching KGE ≥ 0.5 and only 21% falling below 0. The highest skill is found in snow-dominated and maritime regions, reflecting reliable winter precipitation and the model’s representation of snowmelt physics. Both KGE and *r* are notably lower in the arid southwest, particularly at gauges representing larger upstream basins where the gap between modeled and observed flow likely reflects a combination of water regulation, basin-scale heterogeneity, and aridity-related runoff-generation errors.

## Usage Notes

Long-term high-resolution water and energy flux data are necessary to understand and prepare for the impacts of hydroclimatic variability from daily to multidecadal scales amid long-term climatic changes. WHiLD-HM provides the longest daily record to-date for WUS at 4-km (1/24°) resolution that can be used in various applications such as ecological modeling and monitoring, and water resources and food security planning. Technical validation demonstrates that WHiLD-HM performs strongly in several aspects, including its ability to capture regional-scale temporal dynamics, local-scale variability across much of the WUS, and the spatial heterogeneity of the water and energy fluxes.

Table 3 highlights the distinct contribution of WHiLD-HM relative to several widely used hydrometeorological, land-surface, and reanalysis datasets. Existing products provide important capabilities, but each has limitations for long-term, high-resolution hydroclimate analysis over the WUS. The Livneh dataset^15^ provides a long historical record, but it is based on the variable infiltration capacity (VIC) land surface hydrological model^64^ and does not include the same Noah-MP land-surface physics used in WHiLD-HM. Furthermore, the Livneh dataset does not extend through the near-present period and therefore misses much of 2020–2025, a critical interval for evaluating recent hydroclimate extremes and disturbance impacts. This interval includes the 2021 extreme drought, 2023 extreme wetness, and the years following the large 2020–2021 fire seasons, when monitoring post-fire hydrologic and land-surface impacts is especially important. NLDAS-2^16^ provides a useful multi-model land data assimilation framework, but it is coarser and begins in 1979. ERA5 and ERA5-Land^65^ offer globally consistent reanalysis products with long records, but their native resolutions are coarser than WHiLD-HM and may not fully resolve the strong topographic gradients that shape western U.S. snow, runoff, and land-atmosphere interactions. In addition, ERA5 precipitation, while generally improved relative to many alternatives, exhibits documented biases, including over complex terrain in the WUS^66–68^. CONUS404^69^ provides 4-km dynamically downscaled fields over CONUS, but its record begins in 1979 and is primarily designed as a regional hydroclimate reanalysis.

**Table 3:** Comparison of WHiLD-HM with selected hydrometeorological, land-surface, and reanalysis datasets commonly used for WUS hydroclimate applications.

Dataset	Spatial resolution	Temporal coverage	Forcing data/data source	Model physics/approach	Key distinction relative to WHiLD-HM
WHiLD-HM	4 km	1952–2025	Observation-constrained forcing; downscaled reanalysis for pressure, radiation, and wind	Noah-MP v5.0 land surface model	—
Livneh dataset^15^	1/16°, ~6 km	1915–2018	COOP temperature and precipitation; PRISM-scaled precipitation; derived humidity/radiation	VIC hydrologic model	Longer record, but VIC-based and less physically detailed than WHiLD-HM’s Noah-MP outputs.
NLDAS-2^16^	1/8°, ~12–14 km	1979–present	NARR-based forcing with gauge precipitation, PRISM adjustment, radar, and satellite radiation	Noah, VIC, Mosaic, and SAC-SMA LSMs	Multi-model LDAS, but coarser and begins in 1979.
ERA5^40^	~31 km	1940–present	Global atmospheric reanalysis with data assimilation	ECMWF IFS with 4D-Var assimilation and land-surface physics	Long global record, but too coarse for many western U.S. terrain and basin-scale applications.
ERA5-Land^65^	~9 km	1950–present	Downscaled ERA5 forcing with elevation correction	Offline ECMWF land surface model	Higher-resolution global land product, but still coarser than WHiLD-HM.
CONUS404^69^	4 km	Oct 1979–Sep 2021	ERA5 dynamically downscaled with WRF	Convection-permitting WRF/Noah-MP regional model	Same 4-km resolution, but starts in 1979 and is primarily atmospheric reanalysis/downscaling.

WHiLD-HM is created using the Noah-MP land surface model version 5.0^14^. We caution users about limitations associated with the land surface model simulation used to create this dataset due to missing processes (e.g., prognostic vegetation and irrigation) and subgrid parameterizations, which limit the dataset’s ability to capture some interactions between hydrometeorological variability, human interventions with the water cycle, and vegetation responses, thereby introducing uncertainty. Forcing data derived from dynamically downscaled ERA5 can contain uncertainties arising from limitations in both the reanalysis and the WRF model itself, some of which are quantified in ref. ^41^. Hourly forcing data for Noah-MP are generated from daily data by superimposing the time-invariant climatological diurnal cycle derived from the coarser NLDAS-2 dataset^16^. As a result, the dataset is suitable only for daily or coarser temporal analyses and cannot be used to examine changes in the diurnal cycle of energy and water fluxes across decades. Uncertainty in Noah-MP forcing data can propagate into the simulated hydrologic and energy variables. Precipitation uncertainty is likely most important in complex terrain and snow-dominated regions, where station density and gauge undercatch can contribute to SM, runoff, and SWE biases. ERA5-WRF radiation, humidity, and wind uncertainties can influence ET, sensible heat flux, sublimation, and snowmelt, while the transition between PRISM-based and ERA5-WRF-based humidity may introduce temporal inconsistencies in humidity-sensitive variables. We did not conduct a formal ensemble-based uncertainty propagation analysis for this release; however, the validation results provide qualitative guidance on regional uncertainty, including larger ET discrepancies in irrigated and arid regions, larger SM differences in snow-covered and arid regions, and an overall tendency toward SWE underestimation. While the dataset is well suited for analyses of variability, spatial patterns, and relative anomalies, users should consider forcing and model uncertainty when using WHiLD-HM.

## Data Availability

Data used and generated in the study are available from the links below. GLEAM ET: https://www.gleam.eu/, FLUXCOM-X-BASE: https://gitlab.gwdg.de/fluxcom/fluxcomxdata, FLUXCOM: https://www.fluxcom.org/EF-Download/, SMAP: https://nsidc.org/data/spl3smp_e/versions/6, SNODAS: https://nsidc.org/data/g02158/versions/1. nClimGrid-Daily: https://www.ncei.noaa.gov/products/land-based-station/nclimgrid-daily, PRISM: https://prism.oregonstate.edu/, ERA5-WRF: https://wrf-cmip6-noversioning.s3.amazonaws.com/index.html#downscaled_products/reanalysis/era5/, NLDAS-2: https://disc.gsfc.nasa.gov/datasets, U.S. Geological Survey National Water Information System: https://api.waterdata.usgs.gov/ogcapi/v0/openapi#/daily.

## Code availability

Noah-MP source code is obtained from https://github.com/NCAR/noahmp. CaMa-Flood source code is obtained from https://global-hydrodynamics.github.io/.

## References

[1] Census profile: West Region. *Census Reporter* http://censusreporter.org/profiles/02000US4-west-region/.

[2] Belote, R. T. *et al*. Wild, connected, and diverse: building a more resilient system of protected areas. *Ecol. Appl.* **27**, 1050–1056 (2017). 10.1002/eap.1527 28263450

[3] Leung, L. R., Qian, Y. & Bian, X. Hydroclimate of the Western United States Based on Observations and Regional Climate Simulation of 1981–2000. Part I: Seasonal Statistics. *J. Clim.* **16**, 1892–1911 (2003).

[4] Belote, R. T. *et al*. Mapping Conservation Strategies under a Changing Climate. *BioScience* **67**, 494–497 (2017). 10.1093/biosci/bix028 PMC5451290 28584341

[5] Dettinger, M., Udall, B. & Georgakakos, A. Western water and climate change. *Ecol. Appl.* **25**, 2069–2093 (2015). 10.1890/15-0938.1 26910940

[6] Averyt, K. *et al*. Sectoral contributions to surface water stress in the coterminous United States. *Environ. Res. Lett.* **8**, 035046 (2013).

[7] Abatzoglou, J. T., Dobrowski, S. Z., Parks, S. A. & Hegewisch, K. C. TerraClimate, a high-resolution global dataset of monthly climate and climatic water balance from 1958–2015. *Sci. Data* **5**, 170191 (2018). 10.1038/sdata.2017.191 PMC5759372 29313841

[8] Abatzoglou, J. T. Development of gridded surface meteorological data for ecological applications and modelling. *Int. J. Climatol.* **33**, 121–131 (2013).

[9] Thornton, P. K., Ericksen, P. J., Herrero, M. & Challinor, A. J. Climate variability and vulnerability to climate change: a review. *Glob. Change Biol.* **20**, 3313–3328 (2014). 10.1111/gcb.12581 PMC4258067 24668802

[10] Wang, J. *et al*. Association of western US compound hydrometeorological extremes with Madden-Julian oscillation and ENSO interaction. *Commun. Earth Environ.* **5**, 314 (2024).

[11] Cayan, D. R., Redmond, K. T. & Riddle, L. G. ENSO and Hydrologic Extremes in the Western United States. *J. Clim.* **12**, 2881–2893 (1999).

[12] Hidalgo, H. G. Climate precursors of multidecadal drought variability in the western United States. *Water Resour. Res*. **40**, W12504 (2004).

[13] Kuo, Y.-N., Kim, H. & Lehner, F. Anthropogenic Aerosols Contribute to the Recent Decline in Precipitation Over the U.S. Southwest. *Geophys. Res. Lett.* **50**, e2023GL105389 (2023).

[14] He, C. *et al*. Modernizing the open-source community Noah with multi-parameterization options (Noah-MP) land surface model (version 5.0) with enhanced modularity, interoperability, and applicability. *Geosci. Model Dev.* **16**, 5131–5151 (2023).

[15] Livneh, B. *et al.* A Long-Term Hydrologically Based Dataset of Land Surface Fluxes and States for the Conterminous United States: Update and Extensions. *J. Clim.* **26**, 9384–9392 (2013).

[16] Xia, Y. *et al*. Continental-scale water and energy flux analysis and validation for the North American Land Data Assimilation System project phase 2 (NLDAS-2): 1. Intercomparison and application of model products. *J. Geophys. Res. Atmos.* **117**, D03109 (2012).

[17] Mesinger, F. *et al.* North American Regional Reanalysis. *Bull. Am. Meteorol. Soc.* **87**, 343–360 (2006).

[18] Williams, A. P. *et al*. Large contribution from anthropogenic warming to an emerging North American megadrought. *Science* **368**, 314–318 (2020). 10.1126/science.aaz9600 32299953

[19] Cook, B. I. *et al*. Megadroughts in the Common Era and the Anthropocene. *Nat. Rev. Earth Environ.* **3**, 741–757 (2022).

[20] McKinnon, K. A. & Deser, C. The inherent uncertainty of precipitation variability, trends, and extremes due to internal variability, with implications for Western U.S. water resources. *J. Clim.* **34**, 9605–9622 (2021).

[21] Livneh, B. & Hoerling, M. P. The Physics of Drought in the U.S. Central Great Plains. *J. Clim.* **29**, 6783–6804 (2016).

[22] Jiang, Y. *et al*. The Influence of Land-Surface Conditions on the 2020–2021 Western US Drought. *Water Resour. Res.* **61**, e2024WR038124 (2025).

[23] Williams, A. P. *et al*. Anthropogenic Intensification of Cool-Season Precipitation Is Not Yet Detectable Across the Western United States. *J. Geophys. Res. Atmospheres* **129**, e2023JD040537 (2024).

[24] Seager, R. *et al*. Causes of the 2011–14 California Drought. *J. Clim.* **28**, 6997–7024 (2015).

[25] Ross, T., Lott, N., McCown, S. & Quinn, D. The El Nino winter of ’97-’98. https://repository.library.noaa.gov/view/noaa/13823 (1998).

[26] Waananen, A. O., Harris, D. D. & Williams, R. C. *Floods of December 1964 and January 1965 in the Far Western States; Part 1 Description*. *Water Supply Paper* https://pubs.usgs.gov/publication/wsp1866A, 10.3133/wsp1866A (1971).

[27] Shelton, M. L. The 1976 and 1977 Drought in California: Extent and Severity. *Weatherwise* **30**, 139–153 (1977).

[28] Waananen, A. O., Harris, D. D. & Williams, R. C. *Floods of December 1964 and January 1965 in the Far Western States; Part 2 Streamflow and Sediment Data*. *Water Supply Paper* https://pubs.usgs.gov/publication/wsp1866B, (1970).

[29] Niu, G.-Y. *et al*. The community Noah land surface model with multiparameterization options (Noah-MP): 1. Model description and evaluation with local-scale measurements. *J. Geophys. Res. Atmospheres* **116**, D12109 (2011).

[30] Cosgrove, B. *et al*. NOAA’s National Water Model: Advancing operational hydrology through continental-scale modeling. *J. Am. Water Resour. Assoc.* **60**, 247–272 (2024).

[31] He, C. *et al*. Benchmarking and evaluating the NASA Land Information System (version 7.5.2) coupled with the refactored Noah-MP land surface model (version 5.0). *Geosci. Model Dev.* **18**, 8439–8460 (2025).

[32] Xue, Y. *et al*. Advancing NOAA’s Subseasonal and Seasonal Applications and Enhancing Collaboration among Stakeholders, Modelers, and Researchers. *Bull. Am. Meteorol. Soc.* **106**, E1295–E1302 (2025).

[33] He, C. *et al*. *The Community Noah-MP Land Surface Modeling System Technical Description Version 5.0*. https://opensky.ucar.edu/islandora/object/technotes:599, 10.5065/EW8G-YR95 (2023).

[34] Williams, A. P. Gridded 1/24-degree daily climate for the western United States, 1951–2025. *Zenodo* 10.5281/ZENODO.19636837 (2026).

[35] Williams, A. P. *et al*. The Western United States Large Forest-Fire Stochastic Simulator (WULFFSS) 1.0: a monthly gridded forest-fire model using interpretable statistics. *Geosci. Model Dev.* **19**, 1157–1191 (2026).

[36] Durre, I., Arguez, A., Schreck, C. J., Squires, M. F. & Vose, R. S. Daily High-Resolution Temperature and Precipitation Fields for the Contiguous United States from 1951 to Present. *J. Atmospheric Ocean. Technol.* **39**, 1837–1855 (2022).

[37] Durre, I. *et al*. NOAA nClimGrid-Daily Version 1 – Daily gridded temperature and precipitation for the Contiguous United States since 1951. *NOAA National Centers for Environmental Information* 10.25921/C4GT-R169 (2022).

[38] Daly, C. *et al*. Challenges in Observation-Based Mapping of Daily Precipitation across the Conterminous United States. *J. Atmospheric Ocean. Technol.* **38**, 1979–1992 (2021).

[39] PRISM Group at Oregon State University. https://prism.oregonstate.edu/.

[40] Hersbach, H. *et al*. The ERA5 global reanalysis. *Q. J. R. Meteorol. Soc.* **146**, 1999–2049 (2020).

[41] Rahimi, S. *et al*. Evaluation of a Reanalysis‐Driven Configuration of WRF4 Over the Western United States From 1980 to 2020. *J. Geophys. Res. Atmospheres* **127**, e2021JD035699 (2022).

[42] CMIP6 GCMs downscaled using WRF. https://registry.opendata.aws/wrf-cmip6/.

[43] Draxl, C. *et al*. *WTK-LED: The WIND Toolkit Long-Term Ensemble Dataset*. NREL/TP–5000-88457 10.2172/2473210 (2024).

[44] Wang, J., Bodini, N., Purkayastha, A., Young, E. & Draxl, C. WIND Toolkit Long-Term Ensemble Dataset. DOE Open Energy Data Initiative (OEDI); National Renewable Energy Laboratory (NREL) 10.25984/2504176 (2024).

[45] Peco, K. *et al*. Evaluation of a high-resolution regional climate simulation for surface and hub-height wind climatology over North America. *Wind Energy Sci.* **11**, 13–35 (2026).

[46] Allen, R. G., Pereira, L. S., Raes, D. & Smith, M. Crop evapotranspiration - guidelines for computing crop water requirements. FAO Irrigation and Drainage Paper 56 (Food and Agriculture Organization of the United Nations, Rome, 1998).

[47] gridMET. *Climatology Lab* https://www.climatologylab.org/gridmet.html.

[48] Miralles, D. G. *et al*. GLEAM4: global land evaporation and soil moisture dataset at 0.1° resolution from 1980 to near present. *Sci. Data* **12**, 416 (2025). 10.1038/s41597-025-04610-y PMC11894173 40064907

[49] Nelson, J. A. *et al*. X-BASE: the first terrestrial carbon and water flux products from an extended data-driven scaling framework, FLUXCOM-X. *Biogeosciences* **21**, 5079–5115 (2024).

[50] Jung, M. *et al*. The FLUXCOM ensemble of global land-atmosphere energy fluxes. *Sci. Data* **6**, 74 (2019). 10.1038/s41597-019-0076-8 PMC6536554 31133670

[51] O’Neill, P. *et al*. Soil moisture active passive (SMAP) project: Calibration and validation for the L2/3_SM_P version 7 and L2/3_SM_P_E version 4 Data Products. *Pasadena CA USA: Jet Propulsion Laboratory* (2020).

[52] Xia, Y. *et al*. Evaluation of multi-model simulated soil moisture in NLDAS-2. *J. Hydrol.* **512**, 107–125 (2014).

[53] Koster, R. D. *et al*. On the Nature of Soil Moisture in Land Surface Models. *J. Clim.* **22**, 4322–4335 (2009).

[54] Barrett, A. P. *National Operational Hydrologic Remote Sensing Center SNOw Data Assimilation System (SNODAS) Products at NSIDC.* NSIDC Special Report 11 (National Snow and Ice Data Center, Digital Media, Boulder, CO, USA, 2003).

[55] Falcone, J. A. *GAGES-II: Geospatial Attributes of Gages for Evaluating Streamflow*. https://pubs.usgs.gov/publication/70046617, 10.3133/70046617 (2011).

[56] He, C. *et al*. Can Convection-Permitting Modeling Provide Decent Precipitation for Offline High-Resolution Snowpack Simulations Over Mountains? *J. Geophys. Res. Atmospheres* **124**, 12631–12654 (2019).

[57] Yamazaki, D., Kanae, S., Kim, H. & Oki, T. A physically based description of floodplain inundation dynamics in a global river routing model. *Water Resour. Res*. **47**, W04501 (2011).

[58] Yamazaki, D. *et al*. A high‐accuracy map of global terrain elevations. *Geophys. Res. Lett.* **44**, 5844–5853 (2017).

[59] Yamazaki, D. *et al*. MERIT Hydro: A High-Resolution Global Hydrography Map Based on Latest Topography Dataset. *Water Resour. Res.* **55**, 5053–5073 (2019).

[60] USGS Water Data for the Nation. https://waterdata.usgs.gov/nwis/.

[61] Gupta, H. V., Kling, H., Yilmaz, K. K. & Martinez, G. F. Decomposition of the mean squared error and NSE performance criteria: Implications for improving hydrological modelling. *J. Hydrol.* **377**, 80–91 (2009).

[62] Nash, J. E. & Sutcliffe, J. V. River flow forecasting through conceptual models part I — A discussion of principles. *J. Hydrol.* **10**, 282–290 (1970).

[63] Madakumbura, G. *et al*. WHiLD-HM: A 4-km long-term (1952–2025) daily hydrometeorological dataset for the western United States. *Harvard Dataverse* 10.7910/DVN/X7EYAN (2026). PMC13662183 42350453

[64] Liang, X., Lettenmaier, D. P., Wood, E. F. & Burges, S. J. A simple hydrologically based model of land surface water and energy fluxes for general circulation models. *J. Geophys. Res. Atmospheres* **99**, 14415–14428 (1994).

[65] Muñoz-Sabater, J. *et al*. ERA5-Land: a state-of-the-art global reanalysis dataset for land applications. *Earth Syst. Sci. Data* **13**, 4349–4383 (2021).

[66] Akinsanola, A. A., Jung, C., Wang, J. & Kotamarthi, V. R. Evaluation of precipitation across the contiguous United States, Alaska, and Puerto Rico in multi-decadal convection-permitting simulations. *Sci. Rep.* **14**, 1238 (2024). 10.1038/s41598-024-51714-3 PMC10786863 38216703

[67] Lavers, D. A., Simmons, A., Vamborg, F. & Rodwell, M. J. An evaluation of ERA5 precipitation for climate monitoring. *Q. J. R. Meteorol. Soc.* **148**, 3152–3165 (2022).

[68] Tarek, M., Brissette, F. P. & Arsenault, R. Evaluation of the ERA5 reanalysis as a potential reference dataset for hydrological modelling over North America. *Hydrol. Earth Syst. Sci.* **24**, 2527–2544 (2020).

[69] Rasmussen, R. M. *et al*. CONUS404: The NCAR–USGS 4-km Long-Term Regional Hydroclimate Reanalysis over the CONUS. *Bull. Am. Meteorol. Soc.* **104**, E1382–E1408 (2023).

